# Local Applications

**Published:** 1888-03

**Authors:** D. C. McLaren

**Affiliations:** Nashville, Mich.


### LOCAL APPLICATIONS.

D. C. McLaren, M. D., Nashville, Mich.

The local exhibition of drugs, homœopathically indicated, is a subject that seems to have been neglected almost entirely by the writers of our school. This judgment is based not only on extensive reading, but also upon observation of the practice of not a few fairly good homœopaths, who still cling to allopathic methods and measures in this important respect. A goodly number reject medicated dressings *in toto*, and while theoretically they have, no doubt, the best of the argument, there are enough good and sufficient reasons to bias practical physicians and surgeons in favor of such dressings. Briefly stated, these reasons are as follows: Firstly, the comfort of the patient; secondly, the mental effect on the patient and attendants; thirdly, the possibility of thus administering a second remedy in a low potency for some troublesome local symptom, without interfering with the action of a previously administered internal remedy; and fourthly, the still rarer possibility of thus finding the most fitting simillimum, *e. g.*, the indicated remedy having failed to cure in repeated doses of differing potencies, the same remedy may possibly cure if exhibited locally. While this forms, to my mind, a valid argument, I grant that it is largely problematical, the only instance in point being the case related by Dr. P. P. Wells, at Saratoga, of a prompt cure by holding a powder of the indicated remedy in the hand, the same drug having been taken in vain for years.

An exception may be taken to my third reason. I will illustrate it by the recital of a case. A young wife was taken with menorrhagia from overexertion; there being considerable fever, with flushed cheeks, headache, full, bounding pulse, bearing-down pains, and flow of bright red blood, the indicated *Belladonna* was given in a single dose, and the patient improved rapidly. A day or two after, she complained of matter (leucorrhœa) escaping from her, which so burnt and irritated the parts that her only comfort lay in continued cleansings. Strictly speaking, this did not form a new indication, as the first remedy was doing its work well, and if uninterrupted might cure this untoward symptom also; but having an impatient husband to deal with, I forthwith made up an injection of *Silicea*$^{6x}$ 10 minims to a half ounce of alcohol, a teaspoonful in sufficient water for the purpose. This gave prompt relief from the local trouble, and allowed the general improvement to go on undisturbed.

A fifth argument for homœopathic local applications may be found in the fact of the exceeding sensitiveness of raw and sore surfaces, as affording excellent media for dry administration. How brutally this knowledge is used by the allopath we all know.

The question of potency is one deserving consideration. Until recently all my local applications had been in the crude form as tinctures or infusions, but I was led to try local applications of the lower potencies through a recommendation to that effect in H. N. Guernsey's *Obstetrics.* The results have been so good as to induce me to go further in this direction, and besides the logic of the position is irresistible: If the effective, penetrating force of drugs is heightened in activity by potentization, the necessity for the use of such potencies externally is greater, if that be possible, than the same internally. I will now endeavor to give as concisely as possible a list of remedies, with their homœopathic usefulness externally, with the understood proviso throughout, that other symptoms must agree, and that the topical application of a drug must be supplementary to its internal use, not substitutive.

ACETIC ACID.—Burns and scalds. Stings and bites of animals, even rabid.

After bruises and sprains, when dry heat follows.

VINEGAR.—*Apply freely to lips and nostrils in cases of prolonged unconsciouness from all anœsthetic vapors, and for subsequent drowsiness, dizziness, headache, etc.*

ACONITE.—Burns from alkalies.

Apply compress wet with potency to forehead in cases of sunstroke from direct exposure. Eye wash in inflammation from foreign bodies, cold, and suppressed gonorrhœa. Enema for ascarides; also after each stool in infantile chopped-spinach diarrhœa. As a surgical dressing in grave cases of injury with internal congestions (C. Hg.)

ÆSCULUS.—Anal and hemorrhoidal troubles, as anema, wash or unguentum.

AGNUS CASTUS.—Sometimes indicated in bruises and wounds, sprains, dislocations, and strains from overlifting.

Prevents excoriation from walking. Wash for itching ulcers.

AMMONIA CARB.—Rhus poisoning. Insect stings.

ANACARDIUM.—Rhus poisoning.

ARNICA.—The first application to be made in all cases of traumatism; for this purpose an infusion of the flowers made at the time will be found most generally useful; the injured part may be immersed in a pail or pan of tepid water colored with the infusion. In cases of central concussion, use freely as a wash or douche, or even a bath.

After evacuation of cold abscess, burrowing pus, etc., inject watery solution to prevent further suppuration and septicæmia, varicose and painful ulcers, small painful boils, painful corns, insect stings and bites of rabid or angry animals.

ARSENIC.—Old ulcers, carbuncles, open cancers with agonizing burning pain; a trituration in water or applied dry to the part may give considerable relief and supplement the internal action of the drug very satisfactorily.

BELLADONNA is frequently applied to boils indiscriminately. Should be used only when indicated, and then a potency will be found more efficacious than the tincture.

CALENDULA.—Injuries with destruction of tissue; wounds made by machinery; in lacerated perineum it is invaluable, as with Arnica; keep the flowers on hand and make a fresh infusion when needed.

CANTHARIS.—In burns of the first degree, before blisters have formed, painting with a low potency will give immediate relief and prevent the bullæ rising. In more severe burns and scalds use higher potency in water, as a soothing and healing dressing. Said to have removed freckles, but this cannot be vouched for.

CARB. ANIMALIS.—Painful, stringy scars, erysipelatous swellings, swollen glands, cancers, gummata, with lancinating, cutting, and burning pain. When benign suppurations become offensive.

CROCUS SATIVUS.—Painful suppuration of bruised parts. Old cicatrized wounds reopen and suppurate.

GRAPHITES.—Old ulcers with fetid pus, proud flesh, itching, stinging. Old scars from ulcers.

HAMAMELIS.—The best known application for slight burns. (Lippe.)

HEPAR.—Unhealthy ulcers, with bloody corrosive discharge, and painful, stinging, burning edges. Also, after injuries, sores and swellings have been painted with Iodine.

HYPERICUM.—Injuries to fingers, toes, and nails. Punctured and lacerated wounds, with great pain. Rat bites: crushed fingers, and whenever the pain of wounds, rheumatism, corns, bunions, etc., is so great as to show injury to the nerves.

KREOSOTUM.—Injection for cancer uteri, with acid, putrid discharges, and painful to slight touch. Hard lump on cervix, painful. In severe burns, dilute Creosote water greatly relieves. (Lippe.)

LACHESIS.—Carbuncle, purple color, and surrounded by small boils. Malignant pustule. Bed sores, with black eyes; scars break open and bleed.

LEDUM.—Punctured wounds from slivers of wood, suppuration following. Stings of insects, especially mosquitoes.

LITHIUM CARB.—Barber's itch. Dry, itching ringworm. Syphilitic rash and crusta lactea.

LIME WATER.—Burns from mineral acids. (Lippe.)

MILLEFOLIUM.—Profusely bleeding wounds, especially from a fall; sprains; soreness from overlifting.

PETROLEUM.—Sprains and bruises, burns and scalds.

PHYTOLACCA.—Barber's itch, ringworm; cancerous and syphilitic ulcers. Gargle in diphtheria and simple angina.

RHUS TOX.—Sprains, chilblains, carbuncles; bluish, gangrenous.

SAPO.—Severe burns, with destruction of tissue. Make a paste of castile soap and spread on linen, at the same time giving *Sapo* internally. If the sore becomes putrid and offensive change remedy and dressing to Kreosote.

SYMPHYTUM.—Black eye and other injuries of facial bones. Fractures in general.

STAPHISAGRIA.—Mechanical injuries from sharp cutting instruments, hence after surgical operations.

TEREBINTHINA.—Burns and scalds of second degree.

URTICA URENS.—Burns, involving only the skin. Much burning, accompanied by itching.

